# Overseas Nursing Association

**Published:** 1920-07-03

**Authors:** 


### Overseas Nursing Association.

MEETING AT NORFOLK HOUSE.

The annual meeting of the Overseas Nursing Association was held, by the kind invitation of Her Grace the Duchess of Norfolk, at Norfolk House, St. James' Square, S.W. 1, on Thursday, June 24th. H.R.H. Princess Beatrice, Patroness of the Association, honoured the meeting by being present. The Right Hon. Viscount Gladstone, G.C.B., G.C.M.G., G.B.E., presided.

Viscount Gladstone, after welcoming H.R.H. Princess Beatrice, said it was very gratifying to know that the Association was in touch with every part of our overseas Empire, although its hope with regard to Canada had not quite been realised, the proposals made to Canada not having met with the approval it had wished for. In speaking of the King Edward's nurses who had been sent out to South Africa, he referred to the appointment of H.R.H. Prince Arthur of Connaught as Governor-General of South Africa, of the work in the nursing world of H.R.H. Princess Arthur, and the invaluable help the Princess would be able to give to the newly born.

The adoption of the report was moved by Sir William Manning, who referred in eloquent terms to the work done in Central Africa by the overseas nurses under most trying conditions in days gone by. The arrival of the first two nursing sisters did much, he said, to improve the housing conditions in Nyasaland. The Overseas Nursing Sisters were a wonderful, devoted body of women; the highest praise was none too great for them.

The report showed that the work of the Association had steadily increased since the cessation of hostilities. An unusually large number of vacancies had occurred abroad, and applications from applicants relieved from military duties and anxious for further work abroad had been numerous. During the year under review 742 candidates had been interviewed, as against 117 in the previous year. Of these, 213 were found suitable for work abroad, as against thirty-four. Twelve nurses had been awarded the R.R.C. in recognition of their services during the war, and recently Mrs. Anna Pordage, matron since January 1914 of the Victoria Hospital, St. Helena, received the Order of M.B.E. for her work during the war. The Committee had been in correspondence with the private branches and the authorities of the private hospitals regarding the adequacy of salaries and allowances to meet present-day expenses, and, where necessary, increases had been made. The income and expenditure account shows a deficit on the year of £130, reducing the accumulated surplus on this account to £635. The adoption of the report was seconded by Miss Pratt, of Uganda, who gave a very vivid and interesting account of the conditions of nursing in Uganda and her experiences in many lands during the last twenty years.

Tea was served at the conclusion of the proceedings.

